# Representative Contact Diaries for Modeling the Spread of Infectious Diseases in Taiwan

**DOI:** 10.1371/journal.pone.0045113

**Published:** 2012-10-03

**Authors:** Yang-chih Fu, Da-Wei Wang, Jen-Hsiang Chuang

**Affiliations:** 1 Institute of Sociology, Academia Sinica, Taipei, Taiwan, Republic of China; 2 Institute of Information Science, Academia Sinica, Taipei, Taiwan, Republic of China; 3 Epidemic Intelligence Center, Centers for Disease Control, Taipei, Taiwan, Republic of China; 4 Institute of Biomedical Informatics & Institute of Public Health, National Yang-Ming University, Taipei, Taiwan, Republic of China; Umeå University, Sweden

## Abstract

Recent studies of infectious diseases have attempted to construct more realistic parameters of interpersonal contact patterns from diary-approach surveys. To ensure that such diary-based contact patterns provide accurate baseline data for policy implementation in densely populated Taiwan, we collected contact diaries from a national sample, using 3-stage systematic probability sampling and rigorous in-person interviews. A representative sample of 1,943 contact diaries recorded a total of 24,265 wide-range, face-to-face interpersonal contacts during a 24-hour period. Nearly 70% of the contacts occurred outside of respondents' households. The most active age group was schoolchildren (ages 5–14), who averaged around 16–18 daily contacts, about 2–3 times as many as the least active age groups. We show how such parameters of contact patterns help modify a sophisticated national simulation system that has been used for years to model the spread of pandemic diseases in Taiwan. Based on such actual and representative data that enable researchers to infer findings to the whole population, our analyses aim to facilitate implementing more appropriate and effective strategies for controlling an emerging or pandemic disease infection.

## Introduction

Many policy implementations for disease control rely on modeling how infectious diseases spread. To increase the effectiveness of such modeling, one of the most comprehensive efforts seeks to collect empirical data by means of large-scale probability surveys and infers the findings to the population upon which a policy is to be implemented. Such a task becomes more urgent for densely populated societies that may be more susceptible to a pandemic outbreak.

In particular, recent studies of infectious diseases have constructed more accurate parameters of interpersonal contact, based on diary-approach surveys from both Europe [1]–[4] and Asia [5]. Other studies have used web-based surveys [6] or time-use methods [7]. To ensure that such diary-based contact patterns provide accurate baseline data for policy implementation, we collected contact diaries from a representative national sample in Taiwan and extracted realistic parameters for our simulation modeling of disease infections.

Using empirical contact data to model how diseases spread has two major policy implications. First, unlike earlier studies, large-scale surveys enable researchers to derive and incorporate more accurate parameters into their models [8]–[9]. Earlier modeling of disease infections, for example, estimated that an average person had contact with either 10 or 20 people at work in a typical day. The estimated number of contacts at school ranged widely from 20, 79, 128 to 155 [10]–[12]. Whether based on simulations or small samples of interviews, such estimates may not reflect overall contact patterns in the whole population. More recent attempts, however, reveal how people actually interact under various circumstances, which helps predict possible trends of disease infections.

Second, diary-based large surveys help distinguish contact patterns not only among various social groups within a population, but also across different countries. Comparative European surveys, for example, have found that residents in Southern Europe have more interpersonal contacts than those in Northern Europe [1]. Another survey revealed fewer contacts among Vietnamese [5]. Such different contact patterns serve to inform researchers and policy makers by helping them model how diseases may spread and rethink how certain intervention strategies may work.

The latest attempts have helped approach the ultimate goal of clarifying how infectious illnesses actually spread among people. A critical point that determines the success of such attempts lies in how rigorously they follow probability sampling and conduct the interviews, both necessary conditions for collecting a sample that can represent the whole population. Recent diary-based large surveys have relied mostly on quota sampling [1]–[2], partly because any systematic sampling of a national population has been inherently sophisticated and the data collection tedious and costly. When a survey's respondents all live in rural areas [5], furthermore, the findings will be less compatible with those of other studies.

Our diary-based survey in Taiwan emphasizes the rigorous requirements on both systematic probability sampling and in-person (face-to-face) household interviews to ensure proper inference to the population as a whole. Only when the modeling of disease infections relies on surveys that target the right populations and apply rigorous procedures in sampling schemes and data collection can policy makers implement effective measures for disease control.

## Methods

### Ethics Statement

Our data were drawn from a survey conducted in 2010 in Taiwan. At the time, no internal review board or research ethics committee was established in Taiwan to review ethics of studies in social and behavioral sciences (pilot research ethics committees for such a purpose are being tested by the National Science Council as of spring 2012). Being fully aware of possible ethics issues involved in our study, however, we took the following steps to protect our survey respondents and anyone mentioned in the survey.

First, we used the personal information from our sample list, as regulated by the law, only to locate and identify our targeted respondents and then destroyed the name list after the interviews. Second, all interviews required respondents' prior written informed consent (or that by the parents of respondents who were under 18 years old). When a targeted respondent was younger than age 9, a parent or a guardian who knew the child well answered the questions on the child's behalf. Third, we kept all completed questionnaires under strict anonymity and confidentiality, left no identifiable personal information (such as names of respondents or anyone listed in the contact diaries) in our electronic data set, and destroyed the completed questionnaires after data cleaning.

### Research Site

As one of Asia's developed economies and emerging democracies, Taiwan presents an interesting case for studies in disease infection. First of all, Taiwan is densely populated (about 634 persons per square kilometer in 2010), yet the infrastructure of public health has been well established [13]–[14]. With its long and extensive experiences with large-scale social surveys, partly facilitated by the comprehensive household and population registration system, Taiwan has conducted the most interviews of any general social survey series in the world [15]. This study follows that tradition in sampling and field interviews, while adding questions about disease infections into the survey designed mainly for 24-hour contact diaries.

Under the influence of the Chinese culture that overly stresses *guanxi* (or relationship) [16]–[17], Taiwan's residents tend to make contact with others often in their everyday lives. Asking questions about interpersonal contacts thus turns out to be significant not only for research on disease infections but also for more general social issues [18]. Under such circumstances, Taiwan offers an opportunity for exploring how rigorous diary-approach surveys may further facilitate researchers' and policy makers' understanding of how infectious diseases spread.

### Survey Design

Fielded in spring 2010, our survey aimed to gain insight into the patterns of interpersonal contact among Taiwan's 23 million residents. The timing of the survey was crucial, given the pandemic H1N1 influenza that caused world-wide concerns in 2009. Taiwan's residents were particularly alert, in the shadow of having endured the most confirmed cases of severe acute respiratory syndrome (SARS) outside of Hong Kong and mainland China during the 2003 outbreak [19]–[20].

The core survey instrument was a 24-hour contact diary log that recorded every face-to-face interpersonal contact the respondent had made during the past 24 hours. To capture the possible channel of air-borne transmission of influenza, we asked respondents to record physical contacts and those nonphysical contacts with verbal communication made within 2 meters. Part of the diary log followed those used in recent studies elsewhere [1]–[5]. This instrument went through a series of modifications after cognitive interviews and a pretest. To ensure that our survey results are representative, we targeted the whole registered population in Taiwan without any age limit. The survey sampling and in-person household interviews were both conducted by the Academia Sinica, Taiwan.

### Sampling and Interviewing

To obtain a representative sample of Taiwanese residents, we followed the sampling procedures used in the well-established Taiwan Social Change Survey (TSCS) [21]–[22]. We first categorized all of Taiwan's 358 towns and cities into 7 strata based on factor analyses of 6 demographic and socioeconomic indices at the town/city level (number of persons per square kilometer, % of college graduates among those over 15 years old, % of those over age 65, % of those aged 15–64, % of those working in the manufacturing sector, and % of those working in the service sector, all taken from census and household registration data). With a goal of collecting 1,900 successful cases (which is more than sufficient for statistical inference and analyses), we followed the rule of “probability proportionate to size” (PPS) to set the number of our targeted respondents in each of these 7 strata, making sure that these numbers were in proportion to the sub-populations.

According to this sampling framework, we used systematic sampling in each of the following three stages to determine our targeted respondents: (1) we first selected 34 out of the 358 towns and cities grouped by strata, (2) from each sampled town/city we then picked 2 villages/precincts, and (3) in each of the 68 villages/precincts, we randomly selected 28 to 86 residents directly (skipping the household sampling), depending on the numbers of targets we set earlier. To achieve our goal in the third stage, the aforementioned institutes asked the Ministry of the Interior to provide a computer file that contained all individuals listed in the Household and Population Register (with names, gender, birth dates, and addresses) in each of the 68 targeted villages/precincts.

Following strict survey standards, no substitute respondents were allowed. As a result, we sampled 4,207 residents as our targeted respondents. These targets ranged from 1.4 to 4.0 times more than the expected successful sample size, varying among different towns and cities according to the average response rates from various nationwide surveys over the past 5 years. A total of 41 trained interviewers visited respondents' residences to conduct face-to-face interviews to collect both individual background information and details about all interpersonal contacts during the past 24 hours. When the interviewers could not reach targeted respondents on the sample list, they were instructed to go back to each address at least three more times (in different time slots and on different days of the week) before giving up on the interview.

### Simulation Modeling with Interpersonal Contact

To examine how the empirical data of interpersonal contact may contribute to modeling, we formulate the findings from our survey and apply them to a pandemic flu simulation system developed at Academia Sinica, Taiwan. The detailed implementation of the simulation system has been described elsewhere [23].

### Extracting Parameters from Contact Patterns

We construct contact patterns and social-mixing groups based on the average number of contacts, duration of each contact, and frequency of contact for all age groups. To facilitate easier modeling, we first convert each category of contact duration into *mean contact duration*. For example, we use 2.5 minutes for “less than 5 minutes,” 12 minutes for “5–19 minutes,” 37 minutes for “15–59 minutes,” 150 minutes for “1 to 4 hours,” and 240 minutes for “more than 4 hours.” For the same reason, we create *mean contact frequency*, a specific fraction for each category of contact frequency, such as 1 for “daily,” 0.5 for “almost daily,” 1.5/7 for “once or twice weekly,” 1.5/30 for “once or twice monthly,” 0.5/30 for “less than once per month,” and 1/365 for “never.” We then calculate *weighted contact duration* by multiplying mean contact duration by mean contact frequency. Next, by dividing weighted contact duration by 1,440 (the number of minutes in a day), we obtain *weighted contact probabilities*. Based on the same principle, we are also able to calculate interpersonal weighted contact probabilities in various social-mixing group settings.

Finally, we reconstruct contact probabilities between age groups under various “contexts” of contact (household, household cluster, etc.) [11], [24]–[25], combining the aforementioned age groups into a simplified classification of age groups. The simplified classification divides all respondents into only 2 groups: Child (ages 0–18) and Adult (age 19 and over). To reveal more details, we also compute contact probabilities for Child 0–4, Child 5–19, and Adult 19–64, respectively.

To perform a comparative analysis of the simulation results with findings from previous studies, we further introduce a scale factor to adjust each of the contact probabilities in the various groups to a reasonable range. The case with which we compare is a simulation model for pandemic influenza in the United States (as reported in Proceedings of the National Academy of Sciences in [11], hereafter PNAS).

Based on these realistic parameters, we perform 100 baseline simulations with a basic reproduction number (*R_0_*) of 1.6 for each of the 2 sets of contact probabilities, and calculate the average daily new clinical cases of all 100 simulated results.

## Results

### Characteristics of Study Samples

After two-and-a-half months of fieldwork, we completed 1,954 successful interviews. The respondents (or their parents or guardians) provided 1,943 contact diaries that recorded every person with whom they had contacted during the past 24 hours (up to a maximum of 40 persons, a cutoff set to limit time and space). The “crude” (or “minimum”) response rate was about 46.4% (or 51.3% after excluding ineligible cases such as wrong addresses, etc.)(The response rates are calculated by international standards, cf. *Standard Definitions*: Final Dispositions of Case Codes and Outcome Rates for Surveys, The World Association for Public Opinion Research, 2011. http://wapor.unl.edu/wp-content/uploads/2011/02/StandardDefinitions2011.pdf). Such results are in line with response rates accomplished by the TSCS and other large-scale surveys in Taiwan in recent years [21]–[22].

Using the 2010 Household and Population Register as the criterion, a test of goodness of fit shows that our targeted sample and Taiwan's total population did not differ in any of the distributions for gender, age groups, or the interaction between gender and age groups (P = 0.831, 0.715, and 0.862, respectively). Our completed sample (N = 1,954) was also evenly distributed between males (50.9%) and females (49.1%), although the age distribution was a little skewed towards both ends. The median age was 37, ranging from 0 to 97, with an interquartile range (IQR) from 19 to 54. To modify for the underrepresented subsamples (for ages 21–40), we used a weighted sample in further analyses.

Respondents were well distributed over all geographic regions and across different urbanization levels (about 13.6% rural and 86.4% urban) that matched the distributions of the total population. The household size averaged 4.5. For those over 18 years old (adults), 56.4% were married, and 37.6% received at least college education. About 37.9% of adults were service-sector workers, and 23.8% were manual workers; others were either students (5.5%) or not working (32.8%).

### Contact Patterns in Everyday Life

Our 1,943 contact diaries recorded a total of 24,265 contacted persons during the past 24 hours. The median age of these contacted persons was 33, with an IQR range from 17 to 48. As among the respondents, young adults (ages 20–29) were also underrepresented among the contacted persons. Furthermore, subsamples at both ends of the age structure were even smaller, indicating that our respondents rarely made contact with the very old and the very young.

Much content of the contact diaries indicates that interpersonal contact often occurs with those in one's immediate social circles. For example, about 25.8% of contacted persons were family or household members, and 36.9% were related to one's school or workplace (Table 1). About two thirds of these persons had contact with the respondents on a daily basis. Nearly one third of the contacts involved a physical contact or lasted over 4 hours. A little more than half of the contacts also took place within 1 km from home.

**Table 1:** Distribution of contacted persons by tie and contact features.

Category	Covariate	Frequency (%)
Relationships	School/workplace	8,947 (36.9)
	Household	6,249 (25.8)
	Neither	9,063 (37.3)
Frequency of contact	Daily	16,116 (66.5)
	Weekly	3,899 (16.1)
	Monthly	1,657 (6.8)
	Less often	1,505 (6.2)
	Never	1,065 (4.4)
Location	Home	7,486 (30.9)
	Workplace	6,033 (24.9)
	School	4,691 (19.3)
	Leisure	1,758 (7.3)
	Public transportation	379 (1.6)
	Others	5,060 (20.9)
Types of contact	Non-physical contact	16,169 (67.0)
	Physical contact	7,966 (33.0)
Distance from home	<1 km	12,786 (52.7)
	1–9 km	7,078 (29.2)
	10–49 km	3,558 (14.6)
	>50 km	841 (3.5)
Duration	<5 minutes	4,526 (18.7)
	5–14 min.	3,326 (13.7)
	15–59 min.	3,916 (16.1)
	1–4 hours	5,378 (22.2)
	>4 hours	7,119 (29.3)

Despite such an indication of contact with strong ties, the contact diaries also reveal extensive contacts with those beyond one's core social networks. In particular, nearly 70% of contacts occurred away from respondents' homes (such as at school, in the workplace, on public transportation, during leisure time, etc.). About 17.4% of contacted persons had maintained infrequent contact with respondents (less often than weekly).

The most common and fundamental indicator used in modeling disease infections has been the average number of persons contacted by an individual or a group. With an upper cut off at 40, the average number of persons in our 24-hour diaries reaches 12.5 (S.D. = 9.3), which is close to that in European countries (13.4) yet much higher than in Vietnam (7.7) [2], [5]. Within Taiwan, most regional differences are not significant (for example, the average number of contacts is 12.4 in the North and 12.3 in the South), except for those living in the more remote East (only 9.5).

**Figure 1:**
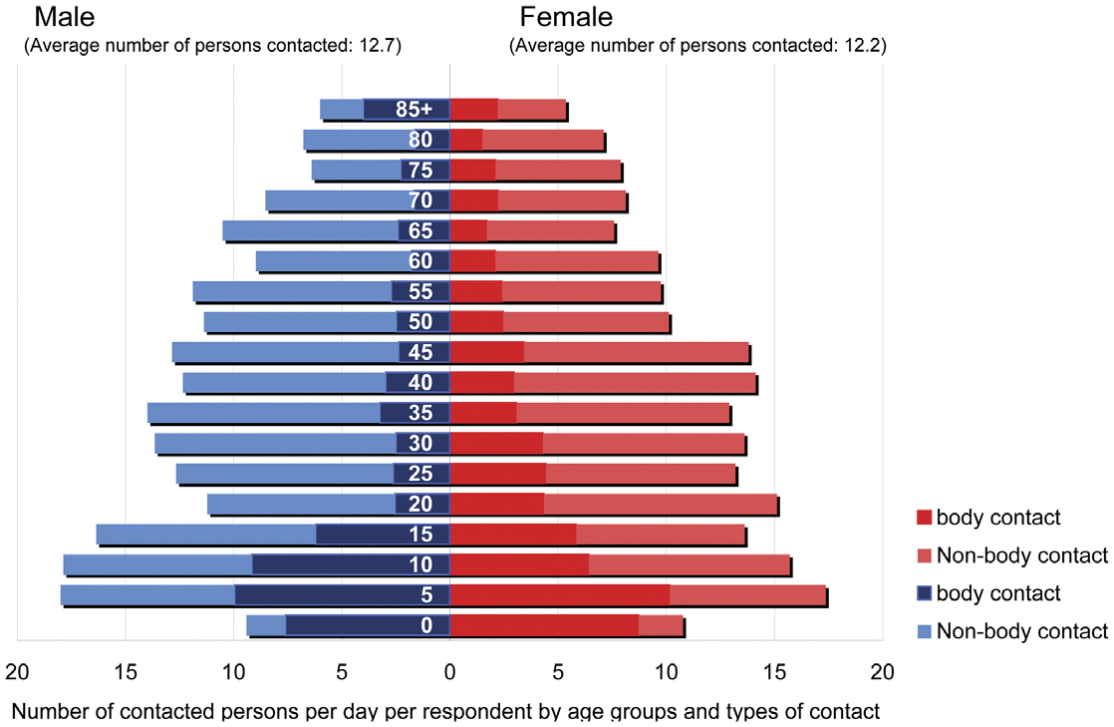
Average numbers of contacted persons by age groups.

As shown in Figure 1, an average male respondent contacts 12.7 persons, only slightly more than the average female respondent (12.2). Age groups, however, vary extensively on how many people are contacted each day. For example, infants, toddlers, and preschoolers (under age 5) have contact with only about 10 persons per day. Those over age 60 also contact fewer than 10. In contrast, middle-aged adults (particularly those in their 30 s and 40 s) seem to be well connected with others through face-to-face contact, although the males in their 20 s are not as active.

The most active age group of all is schoolchildren (ages 5–14), who average around 16–18 contacts. These super spreaders of influenza viruses make 2–3 times as many contacts as the least active age groups [3], [8], [12]. Furthermore, about half of these children's contacts involve a touch or other kind of physical contact.

### A Comparison with the Previous PNAS Study

**Figure 2:**
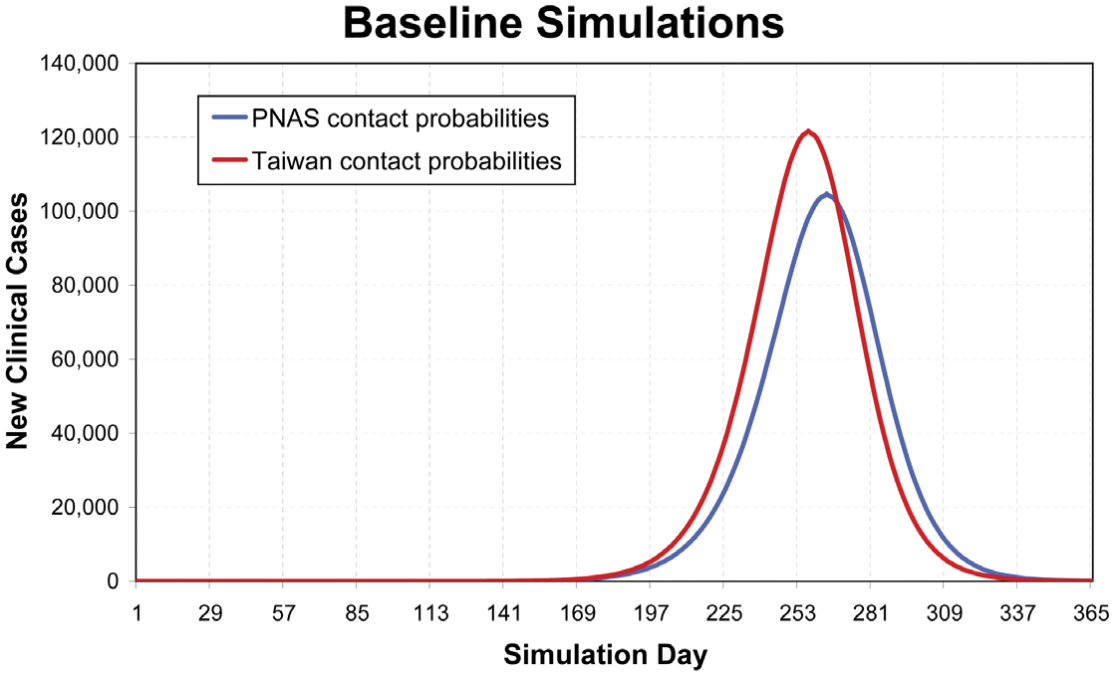
Daily new symptomatic cases of baseline simulations with different sets of contact probabilities.

**Figure 3:**
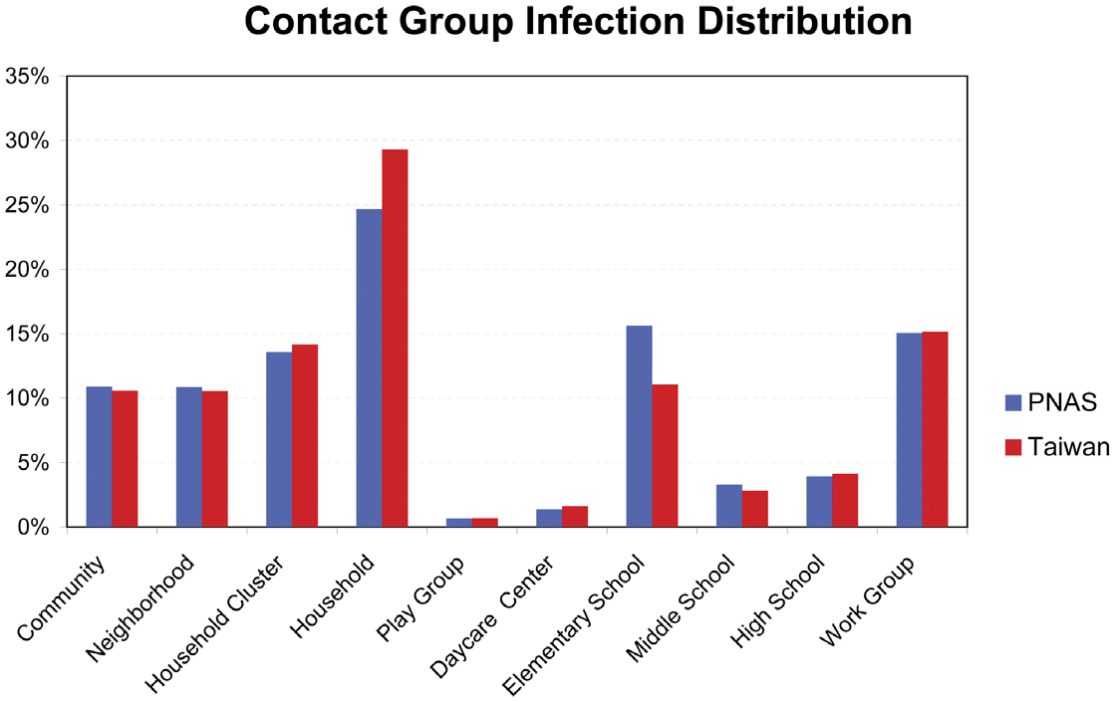
Distribution of infection cases with different sets of contact probabilities.

A comparison of the findings of our study with those of a previous PNAS study in the United States is summarized in Table 2. After implementing the simulations, we can clearly determine that the adjusted contact probabilities would result in a slightly faster and more pronounced epidemic outbreak in Taiwan (Figure 2). A similar pattern can also be observed from the distribution of group infection cases, as shown in Figure 3.

**Table 2:** Comparison of the adjusted social contacts with previous study.

PNAS - contact probabilities for different contact groups				Derived Contact	Scale Factor	Adjusted Contact
Household	Child	Child	0.6	12.61%	4.75	0.60
	Child	Adult	0.3	10.40%	4.75	0.49
	Adult	Child	0.3	10.40%	4.75	0.49
	Adult	Adult	0.4	8.93%	4.75	0.42
Household cluster	Child	Child	0.075	7.30%	1.00	0.073
	Child	Adult	0.04	6.63%	1.00	0.066
	Adult	Child	0.04	6.63%	1.00	0.066
	Adult	Adult	0.05	3.45%	1.00	0.034
Small play group	Child 0–4	Child 0–4	0.35	8.03%	0.40	0.032
Large daycare	Child 0–4	Child 0–4	0.15	8.03%	0.20	0.016
Elementary school	Child 5–18	Child 5–18	0.0435	8.42%	0.40	0.034
Middle school	Child 5–18	Child 5–18	0.0375	8.42%	0.40	0.034
High school	Child 5–18	Child 5–18	0.0315	8.42%	0.40	0.034
Workgroup	Adult19–64	Adult 19–64	0.0575	5.78%	1.00	0.058
Neighborhood	Anyone	Adult 65+	0.00087			0.00087
	Anyone	Adult 19–64	0.00058			0.00058
	Anyone	Child 5–18	0.0002175			0.00022
	Anyone	Child 0–4	0.0000725			0.000073
Community	Anyone	Adult 65+	0.0002175			0.00022
	Anyone	Adult19–64	0.0001450			0.00015
	Anyone	Child 5–18	0.0000544			0.000054
	Anyone	Child 0–4	0.0000181			0.000018

## Discussion and Conclusions

Diary-based surveys help generate realistic parameters for simulating how influenza-like diseases may spread. Being constructed from 24-hour diaries, patterns of interpersonal contact have been particularly helpful in revealing the underlying factors that influence the scope, direction, and speed of disease infections. Because interactions vary by social norms, which often differ from culture to culture, it is essential to design policies based on country-specific empirical data. In pursuit of such a goal, recent attempts at collecting comparative survey data represent a major step towards policy implementations that target the right populations.

To extend these latest efforts, this paper focuses on how to collect and analyze contact diaries from a probability sample that is representative of the national population. We then use the representative contact diaries to construct simulation models. In particular, we model disease infections with matching age groups between infectors (diary keepers) and infectees (contacted persons), and with various occasions of contact.

Our findings suggest that, compared to other studies, the representative contact diaries in Taiwan yielded a wider range of interpersonal contacts. For example, a recent study in rural Vietnam collected 865 contact diaries showing that 85% of all contacts occurred at home, and 93% were with those who kept in touch on a daily basis [5]. With only 30.9% of contacts taking place at home and 66.5% interacting daily in our case, our systematic sampling helped obtain a sample that displays more diverse contact patterns in a highly urbanized society.

Compared with findings from European surveys, however, our study reveals less physical contact even in the private realm, a pattern similar to Vietnam. For example, about 53% of home contacts in Taiwan (45% in Vietnam) were physical, which is significantly lower than in European societies (75%). Of the contacts that took place daily, only 41% involved physical touch, which was nearly identical to the finding in Vietnam (40%) but again much lower than in Europe (60%) [2], [5]. Physical contacts among the Taiwanese were also less common in public realms: About 43% of school contacts and 27% of leisure contacts were physical in Taiwan, compared to 50% for both occasions in Europe [2].

Although contact patterns in various settings imply different risks of disease infection, cross-national variations further help explain how societies differ in influenza infections. As documented in recent studies that used diversified contact parameters for social groups [1]–[6], any efforts to model realistic disease infections should take into account how various groups differ in contact patterns in everyday life. Such variations also play a critical role in simulation modeling.

Our attempt helps modify a sophisticated national simulation system that has been used for years to model the spread of pandemic diseases in Taiwan. The results brought by the new parameters also show noticeable differences from similar modeling based on data from the United States. Given the nature of disease infections, the simple diary-based approach shall continue to be the most comprehensive method to collect details about face-to-face interpersonal contacts in everyday life. The difficulties of data collection may be eased by the use of more technically advanced methods, such as online surveys. Nonetheless, because such surveys tend to undersample technically disadvantaged groups (who may also be more or less vulnerable to disease infections), one should pay special attention to the validity of statistical inferences based on data collected from the use of such methods. More country-specific findings are expected as an increasing amount of comparable diary-based probability survey data becomes available from other countries. For example, preliminary analyses show that the Taiwanese have a different cross-generational pattern of interpersonal contact. While European diary-based studies reveal frequent interactions between children and adults about 30–40 years apart [1]–[4], Taiwan diaries show additional and noticeable interactions between age cohorts that are about 60 years apart.

This latter pattern suggests that the living or parenting arrangements in Asia may differ from those in the West. It is not uncommon in Taiwan for people from three generations to live under the same roof, or for grandparents to help take care of their young grandchildren on a daily basis. In such a circumstance, both care givers (seniors) and care takers (toddlers or young children) may well belong to the age groups that are also most susceptible to influenza infections. Does this special feature of age composition within the household contribute to any divergent cross-cultural patterns of disease infections? When contact patterns in everyday life are subject to such cultural norms, policy makers should make the best use of diary-generated empirical data and design intervention strategies accordingly.

In the wave of facing the global threat by new influenza (such as pandemic H1N1 2009) and other infectious diseases, Taiwan and other countries have implemented more robust infrastructure and nationwide surveillance systems for disease control [26]–[28]. To further strengthen intervention strategies and other policy implementations, it is crucial to apply sociological tools about probability sampling and direct recordings of actual human behaviors via contact diaries.

A critical value of these tools lies in their capability to make statistical inferences from a representative sample to the target population. Although probability sampling has been widely used in large social surveys, our study combines rigorous procedures in both systematic sampling and in-person household interviews with 24-hour diary recording.

Unlike small scale studies of contact diaries that aim to build long-term actual and comprehensive archives of interpersonal contacts [29]–[31], our large-scale probability survey helps generate contact diaries that are short-term yet representative. With actual data and a better understanding of contact patterns, such studies should help efforts to implement more appropriate and effective strategies in controlling an emerging or pandemic disease infection.
